# Osteoarticular lesion in xanthoma disseminatum treated with total hip arthroplasty: a case report

**DOI:** 10.1186/s13256-019-2005-z

**Published:** 2019-03-05

**Authors:** Yu Taniguchi, Tomofumi Nishino, Hisashi Sugaya, Mishima Hajime, Naoyuki Ochiai, Masashi Yamazaki

**Affiliations:** 1 Department of Orthopaedics Surgery, Faculty of Medicine, University of Tsukuba, 1-1-1 Tennodai, Tsukuba, Ibaraki 305-8575 Japan; 2 Division of Regenerative Medicine for Musculoskeletal System, Faculty of Medicine, University of Tsukuba, 1-1-1 Tennodai, Tsukuba, Ibaraki 305-8575 Japan

**Keywords:** Arthrosis, Hip arthroplasty, Xanthoma disseminatum

## Abstract

**Introduction:**

Xanthoma disseminatum is a very rare disease classified as a benign non-Langerhans cell histiocytosis, which is rarely associated with osteoarticular lesions. There is only a report of tumor abrasion during treatment of osteoarticular lesions of this disease, artificial joint replacement has not been reported. We describe a patient in whom bilateral total joint replacement was performed for disseminated xanthoma lesions of the hip joints.

**Case presentation:**

A 34-year-old Japanese woman had a chief complaint of bilateral coxalgia. She had been diagnosed as having disseminated xanthoma. Radiographs showed numerous 5-mm radiolucent bands that resembled worm-eaten tracks in the lower part of the femoral heads adjacent to the joint surface. In addition, short tau inversion recovery imaging scans showed high-intensity areas from the femoral head to the neck in both femurs, suggesting bone marrow edema. Total hip arthroplasty was performed for hip arthrosis on both hip joints caused by disseminated xanthoma. Deflection of the implants was a concern from the early stages postoperatively, but both the imaging and clinical findings have been satisfactory for 4 years of follow-up.

**Conclusions:**

A very unusual hip joint lesion of xanthoma disseminatum was replaced with a total artificial joint replacement, and the course over 4 years was good. Our patient’s course will continue to be followed carefully.

## Introduction

Xanthoma disseminatum is a very rare disease classified as a benign non-Langerhans cell histiocytosis, and it is uncommon for the lesion to spread inside a bone or joint [1, 2]. In this report, we describe our experience with a patient who underwent total joint replacement for bilateral hip arthrosis caused by xanthoma disseminatum.

## Case presentation

A 34-year-old Japanese woman had a chief complaint of bilateral coxalgia. She visited the Department of Dermatology at our hospital at 17 years of age after developing yellow-brown papules on her neck, eyelids, and armpits at the age of 16 years. She was diagnosed as having xanthoma disseminatum, and she has been followed up by staff in the Departments of Dermatology and Internal Medicine since then. At the age of 33 years, she developed left coxalgia and visited our department for the first time. Her Japanese Orthopaedic Association (JOA) score of hip joint function was 56 points. Radiographs revealed slight narrowing of the joint space, which manifested as mild arthrosis, but we decided to perform a conservative course of observation. Her left coxalgia became aggravated, and she developed pain in her right hip joint that interfered with activities of daily living (ADLs). Thus, she was hospitalized for close examination and treatment at the age of 34 years.

Regarding her medical history, there was nothing in particular to note apart from xanthoma disseminatum and its complications. Concurrent diseases of xanthoma disseminatum included xanthomas in the hypophysis, respiratory tract mucosa, bulbar conjunctiva, and kidney peripheries, in addition to diabetes insipidus, chronic renal failure, and hypothyroidism. She had undergone tracheotomy at the age of 31 years because of respiratory tract constriction caused by a respiratory tract mucosal lesion. Xanthoma disseminatum had been controlled with orally administered prednisolone.

She experienced pain in both hip joints during walking and body movements, and she was able to walk continuously for approximately 15 minutes. Ranges of motion of both hip joints were restricted to 100°/100° in flexion and 10°/10° in abduction. The JOA scores were 48 in her right hip and 42 in her left hip.

**Fig. 1:**
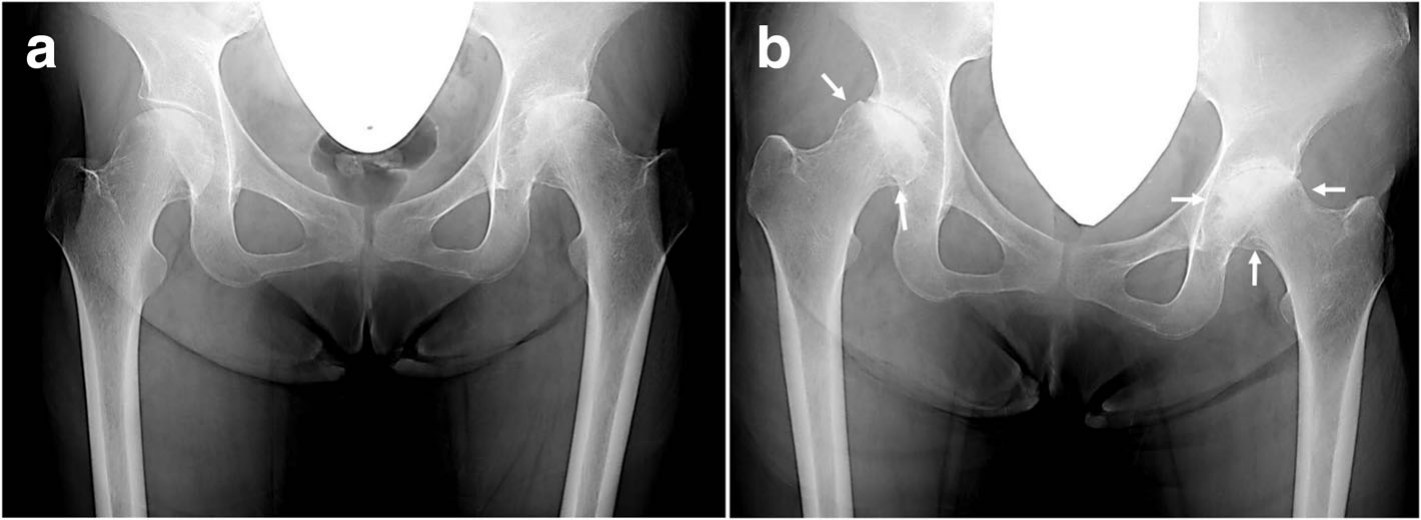
Plain radiographic findings. **a** Initial examination: The joint space in the left hip joint is narrowed with visible irregularities on the loading plane of the head and bone sclerosis. Mild narrowing of the joint space is also seen in the left joint. **b** Upon admission: The joint space in the left hip joint has disappeared and sclerosis on the loading plane has become more severe. Crushing of the femoral head and patterns of radiolucent bands (*arrowed*) resembling worm-eaten tracks are also visible. The joint space has also disappeared on the right side with sclerosis on the loading plane. Crushing of the femoral head has progressed further on the right side than on the left side

**Fig. 2:**
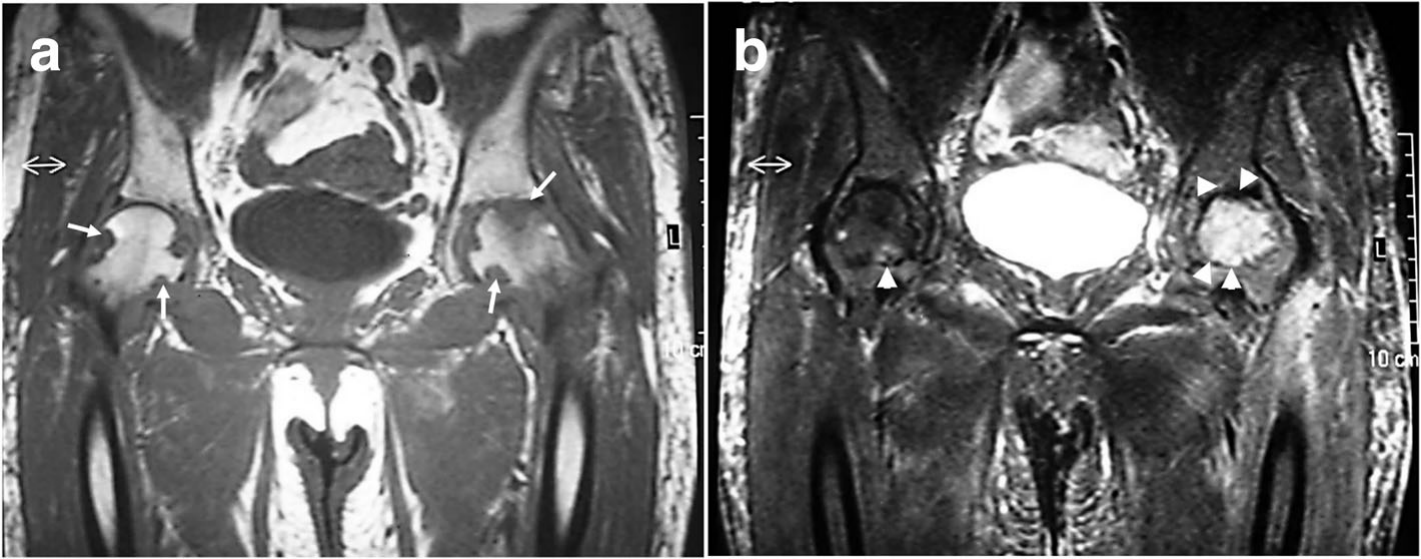
Magnetic resonance imaging findings at the time of admission. **a** T1-weighted image: Lesions with a slightly higher intensity than muscle are observed along the joint capsule and synovial capsule (*arrows*), and tumorous lesions are infiltrating into the bone in a worm-eaten pattern. **b** Short tau inversion recovery image: High-intensity signal (*arrowheads*) in the left femur suggests bone marrow edema

Although plain radiographs revealed narrowing of the joint space, irregularity on the loading plane, and bone sclerosis in her left hip during the first examination, the joint spaces had disappeared in both hip joints by the time of admission, with deformation of the femoral heads, indicating progression of arthrosis. Numerous 5-mm radiolucent bands that resembled worm-eaten tracks were observed in the lower part of the femoral heads adjacent to the joint surface (Fig. 1a, b). A magnetic resonance imaging (MRI) scan revealed high-intensity areas—slightly higher than T1-weighted image intensity—along the joint capsules and synovial capsules that infiltrated the bone in a pattern resembling worm-eaten tracks. Short tau inversion recovery imaging scans showed high-intensity areas from the femoral head to the neck in both femurs—particularly in the left femur—suggesting bone marrow edema (Fig. 2a, b).

Since hip arthrosis progressed rapidly in both hips 1 year after the first examination, we suspected hip arthrosis caused by xanthoma disseminatum. Total hip arthroplasty (THA) was performed on her left hip first because she had more severe pain on the left side. THA was performed on her right hip 9 months later.

**Fig. 3:**
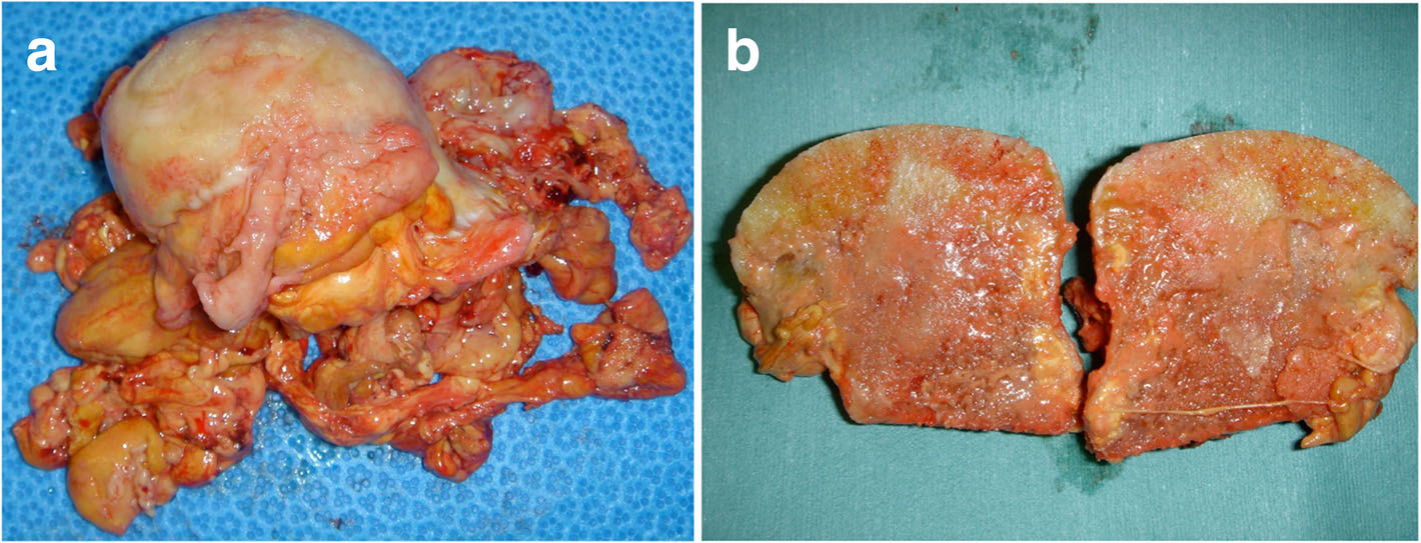
Macroscopic findings of the excised specimen. **a** The left femoral head to neck area is excised. Multiple yellow, tumorous lesions are present around the neck, obscuring the boundary with the joint capsule. **b** A section of the right femoral head: Yellow, tumorous lesions are infiltrating the bone in worm-eaten patterns

A posterolateral approach was used in both hip joint operations, and AMS cups and Perfix 910 stems (both cementless) (Kyocera, Osaka, Japan) were used as implants. The intraarticular pressure was high on both sides, and yellow tumorous lesions bulged from the joint capsule upon incision. The tumorous lesion was excised as much as possible after osteotomy of the femoral neck. Multiple yellow, tumorous lesions in the neck of the femoral heads were dissected with an obscure boundary to the joint capsule. When the femoral head was cut longitudinally, yellow, tumorous lesions infiltrated in patterns of worm-eaten tracks, as observed by radiographic and MRI scans (Fig. 3a, b).

**Fig. 4:**
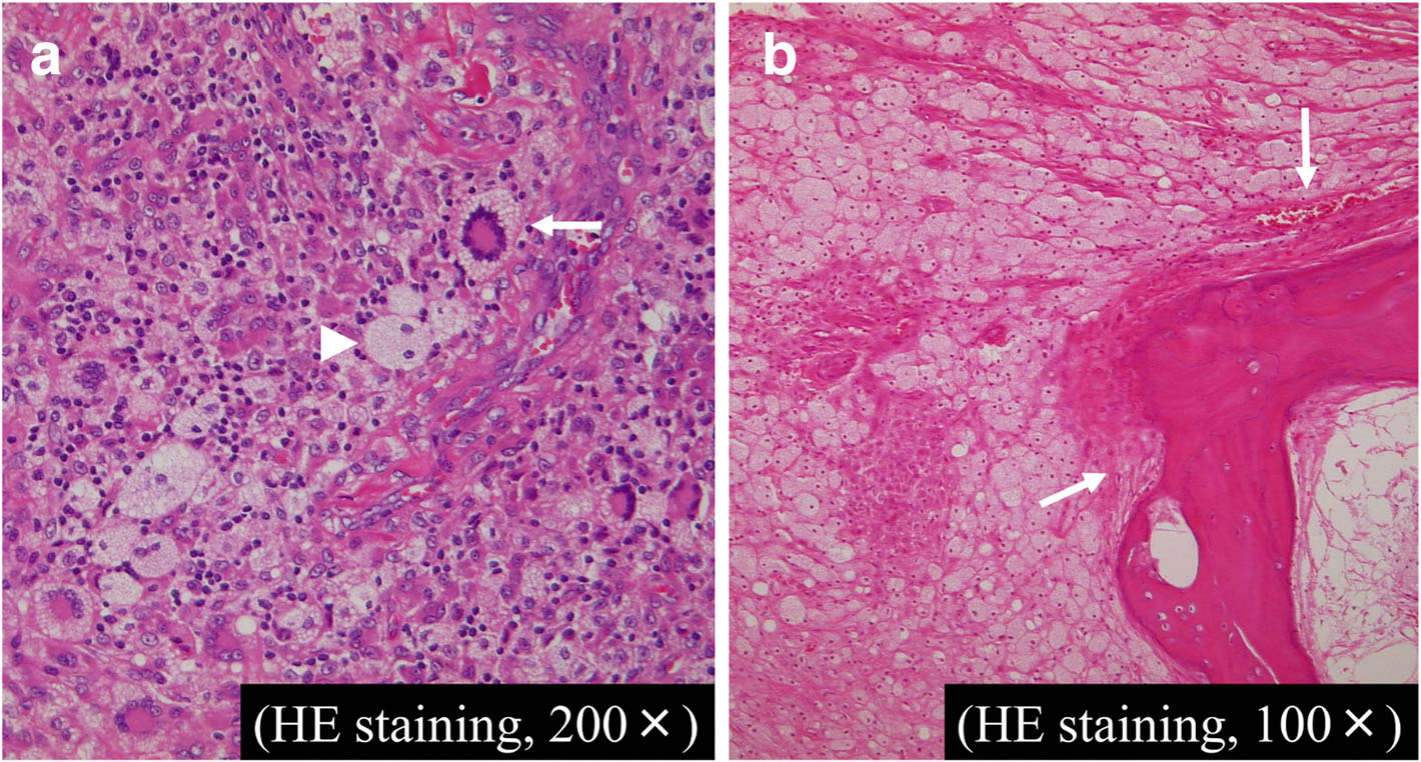
Histopathological findings. **a** Various inflammatory cells, including numerous foamy cells (*arrowheads*), are infiltrating the bone, and Touton-type multinucleated giant cells (*arrows*) are observed. **b** Bone invasion is observed macroscopically, which is indicated by numerous foamy cells and infiltration of histiocyte-like cells (*arrows*). *HE* hematoxylin and eosin

Various inflammatory cells, including xanthoma cells, infiltrated the bone, and Touton-type multinucleated giant cells were observed. Mitosis—observed sporadically in the tumor cells—was normal (Fig. 4a, b). Macroscopic bone defects showed corresponding histological findings, and intraosseous and intraarticular lesions of xanthoma disseminatum were diagnosed.

**Fig. 5:**
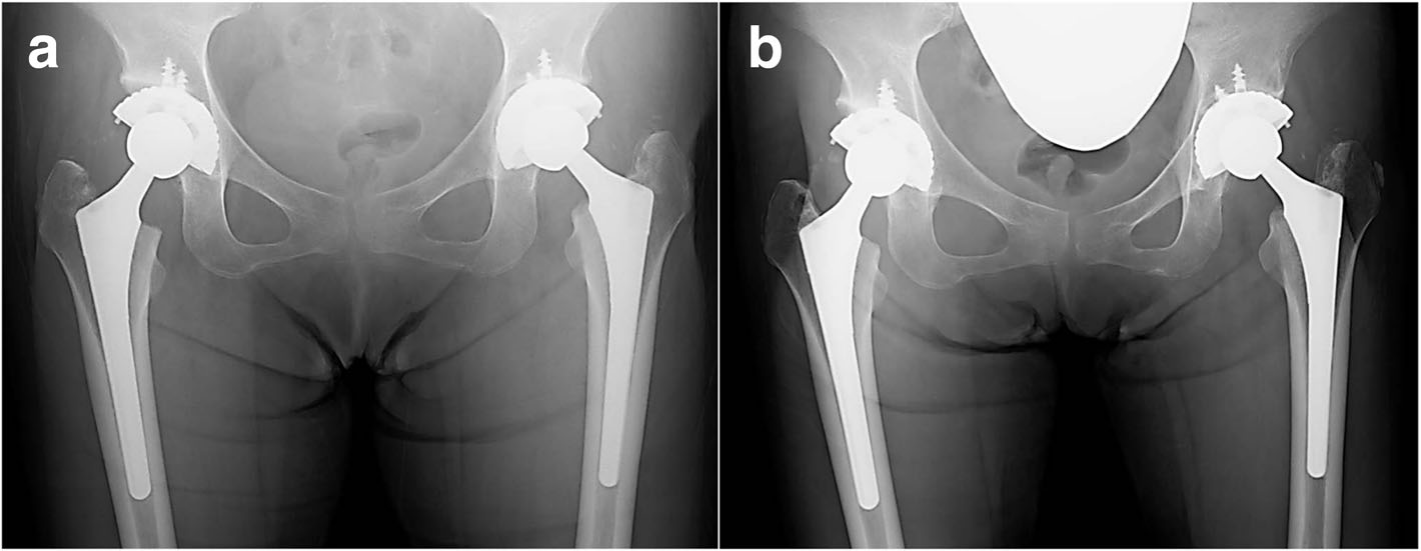
Postoperative plain radiographic findings of the time course of changes. **a** Post-total hip arthroplasty: 1 year after operation of the left hip and 2 months after operation of the right hip. **b** Post-total hip arthroplasty: 4 years and 9 months after operation of the left hip, and 4 years after operation of the right hip. Neither deflection of the implants nor bone defect is noted

At the time of the last observation (4 years after THA of our patient’s right hip joint and 4 years and 9 months after THA of her left hip joint), the JOA scores were 88 in both joints, and there was no disturbance of ADLs. Plain radiographs showed no deflection of the implants, and her postoperative course was satisfactory (Fig. 5a, b).

## Discussion

Xanthoma disseminatum is a rare form of normolipidemic, mucocutaneous xanthoma unaccompanied by hyperlipidemia; reactive granulomatous proliferative disorder of reticuloendothelial histiocytes is considered the cause [3, 4]. The condition was first reported in 1938 by Montgomery and Osterberg, and approximately 100 cases have been reported to date [5]. The age of onset varies widely from 1 to 70 years, but two-thirds or three-quarters of patients develop the disease before the age of 25 years. The disease is characterized by multiple occurrences of yellow to red-brown papulae or nodes in the flexor sides and intertriginous areas of the limbs [2]. Mucosal lesions in the larynx, pharynx, trachea, eardrum, cornea, and other areas have been reported as complications in 31–40% of patients, and diabetes insipidus is a complication in 25–40% of patients [6]. On histological examination, the disease is characterized by xanthogranuloma with foamy cells, Touton-type giant cells, and histiocyte-like cells. Local therapies, such as electrocoagulation, liquid nitrogen cryotherapy, radium patches, X-ray irradiation, surgical excision, and oral administration or intramuscular injection of adrenal cortical hormones, and systemic therapy with oral administration of clofibrate have been attempted with mixed effects; none of these treatments is reliable [7]. A rash may develop after a chronic course, but the prognosis is good [7].

Osteoarticular lesions present rarely, such as in our patient and four additional patients described in the literature [8–11]. Artificial joint replacement has not been reported, as far as we know. Tumor abrasion in the elbow joint was performed in one patient [8], but the therapeutic method was not mentioned in the other reports. In our patient, THA was performed because the clinical symptoms became quickly aggravated, and arthrosis deformans accompanied by infiltration of tumorous lesions into the bones was observed on a plain radiograph and MRI scan. In osteoarticular lesions of disseminated xanthoma, periarticular bone destruction progresses and extends to multiple joints. Early-stage deflection of the implants was anticipated, but adherence has been good thus far.

Pigmented villonodular synovitis (PVS) [12] is a fibrohistiocytic lesion similar to the lesion in our patient. According to Yoo *et al.*, concerning joint replacement for an intraarticular tumor, two of eight patients with PVS who underwent cementless THA required re-replacement [13]. The time to re-replacement was 6.6 years in one patient and 11.3 years in the other patient; osteolysis developed around the acetabular femoral component in both patients. In this report, deflection occurred in patients with PVS who underwent cementless THA at a higher incidence and at an earlier stage than those who underwent normal THA; therefore, we plan to follow-up with our patient carefully.

## Conclusion

Xanthoma disseminatum is a very rare disease classified as a benign non-Langerhans cell histiocytosis, which is rarely associated with osteoarticular lesions. In this case, THA was performed for bilateral hip arthritis associated with xanthoma disseminatum, and the course over 4 years was good. Furthermore, it is necessary that we observe the course of this case.

## Abbreviations

ADLs: Activities of daily living
JOA: Japanese Orthopaedic Association
MRI: Magnetic resonance imaging
PVS: Pigmented villonodular synovitis
THA: Total hip arthroplasty
